# Children’s Eating

**Published:** 1860-10

**Authors:** 


### CHILDREN'S EATING.

SOME parents compel their children to eat against their will, as when they come to the breakfast-table without an appetite, or have lost it in prospect of a visit, or ride, or of going abroad, or for the sake of "eating their plates clean," in discouragement of wasteful habits. Certainly, a child ought to have the privilege of a pig, that of eating only when it is hungry. Unless we are thirsty, we can not drink the purest spring water without a feeling of aversion; and as for eating when there is no appetite, it is revolting; as any one may prove to himself by attempting to take a second meal in twenty minutes after having eaten a regular dinner. The complicated machinery of man, like that of the steam-engine which is in incessant motion, is wearing away every second of his existence. The engine wears out eventually, and a new one has to be constructed; but the machinery of the human body was made by an omnipotent Architect; made to last for ages; made to make its own repairs, to supply its own wastes, so that while it is wearing itself out, it is at the same time regenerating and renewing itself. When the human system is not interfered with, its supply is always equal to its waste: regulated by an unerring instinct—that instinct is called "appetite"—which is greater or less, according to the previous waste; that waste is always in proportion to the exercise which has been taken, as the wear of any machinery is in proportion to its running. Every man knows for himself, that if he walks ten miles he becomes hungry; if fifteen, he is more so. But what makes hunger, and what regulates it to more or less? The wastes of the system set in motion certain processes by which a fluid is prepared, called the gastric juice, and it is so arranged by Divinity, that a certain amount of waste occasions a certain amount of gastric juice; their proportion is exact and uniform; for nature makes no mistakes, does nothing in vain; she makes no more gastric juice than will digest food enough to make up for the waste and want of the body. The appetite, the hunger is excited by the presence of the gastric juice about the stomach; but if there is no gastric juice there can be no hunger, no appetite, and to compel a child to swallow food into the stomach when there is no gastric juice there to receive it, is an absurdity and a cruelty, because, there being no gastric juice there to receive and take care of it, it is rejected by vomiting, or remains there for hours like a "load," or "weight," or "ball," or "heaviness," or else to ferment, causing "oppression," "wind," "acidity," or general discomfort, sometimes for half a night! Similar results take place in old and young, when more food has been taken than there is gastric juice to manage properly; hence, the more than folly of "forcing" food, of eating to "make it even," or taking a single swallow beyond the actual calling of the appetite, expressed in the familiar term, "over-eating," of which too many are conscious, almost every day of their existence. It ought to bring the blush of shame to every cheek, the twinge of penitence to every conscience, because it is a violence offered to the body, a shock imparted to the system, which never fails of more or less derangement, and not unfrequently arrests the machinery of life, to run no more forever! as in an attack of cramp-colic, or deadly diarrhea, (cholera,) at midnight, as a consequence of a late over-hearty meal, or the still more terrible apoplexy, which hurries from life to the judgment without the opportunity of a moment's consciousness, or of the final farewell to the loved ones left behind.

